# Measuring the Burden of Hospitalization in Patients with Parkinson´s Disease in Spain

**DOI:** 10.1371/journal.pone.0151563

**Published:** 2016-03-15

**Authors:** Ruth Gil-Prieto, Raquel Pascual-Garcia, Jesus San-Roman-Montero, Pablo Martinez-Martin, Javier Castrodeza-Sanz, Angel Gil-de-Miguel

**Affiliations:** 1 Area of Preventive Medicine & Public Health, Rey Juan Carlos University, Madrid, Spain; 2 Catedra de Evaluación de Resultados en Salud, Rey Juan Carlos University, Madrid, Spain; 3 Area of Medicine, Rey Juan Carlos University, Madrid, Spain; 4 National Center of Epidemiology and CIBERNED, Carlos III Institute of Health, Madrid, Spain; 5 Department of Preventive Medicine & Public Health, Valladolid University, Valladolid, Spain; University of Pennsylvania Perelman School of Medicine, UNITED STATES

## Abstract

**Introduction:**

This epidemiological survey estimates the hospitalization burden related to Parkinson´s Disease in Spain.

**Methods:**

This observational retrospective survey was performed by reviewing data from the National Surveillance System for Hospital Data, which includes more than 98% of Spanish hospitals. All hospitalizations of patients with Parkinson´s disease that were reported from 1997–2012 were analyzed. Codes were selected using the 9th International Classification of Diseases: ICD-9-CM: 332.0.

**Results:**

A total of 438,513 hospital discharges of patients with Parkinson´s Disease were reported during the study period. The annual hospitalization rate was 64.2 cases per 100,000. The average length of hospital stay was 10 days. The trend for the annual hospitalization rate differed significantly depending on whether Parkinson´s disease was the main cause of hospitalization (n = 23,086, 1.14% annual increase) or was not the main cause of hospitalization (n = 415,427, 15.37% annual increase). The overall case-fatality rate among hospitalized patients was 10%. The case fatality rate among patient´s hospitalized with Parkinson´s disease as the main cause of hospitalization was 2.5%. The hospitalization rate and case-fatality rate significantly increased with age. The primary causes of hospitalization when Parkinson´s disease was not coded as the main cause of hospitalization were as follows: respiratory system diseases (24%), circulatory system diseases (19%), injuries and poisoning, including fractures (12%), diseases of the digestive system (10%) and neoplasms (5%). The annual average cost for National Health Care System was € 120 M, with a mean hospitalization cost of €4,378.

**Conclusions:**

Parkinson´s disease poses a significant health threat in Spain, particularly in the elderly. While hospitalizations due to Parkinson´s Disease are relatively stable over time, the number of patients presenting with Parkinson´s disease as an important comorbidity has increased dramatically. Medical staff must be specifically trained to treat the particular needs of hospitalized patients suffering from Parkinson´s disease as an important comorbidity.

## Introduction

Parkinsonism is a clinical syndrome that is characterized by hypokinesia, tremor, rigidity, and postural instability. Parkinson's disease (PD) is the most common form of parkinsonism, affecting approximately 4.5 million people 50 years of age or older in the most populous nations, including Western Europe. These figures will double to between 8.7 and 9.3 million by 2030 [1]. Although parkinsonisms are not lethal diseases themselves, a modest increase in mortality has been observed in patients with a disease duration beyond 10 years compared with the general population [2,3], and Parkinson´s disease is known to increase hospital admission rates, as well as health care costs [4,5].

Depression, anxiety, sexual dysfunction, sleep disorders, urinary incontinence or retention, constipation, complications derived from medications or dysphagia during the later stages of the disease are common complications in PD. Neuropsychiatric symptoms are well-described non-motor features of PD. Underdiagnosed and often undertreated, they substantially affect the lives of patients and their caregivers. [6].

The impact of Parkinson´s disease (as a comorbid diagnosis) on the burden of hospitalization due to other causes has led to much discussion about recommending its inclusion in a reviewed Charlson Comorbidity Index [7]. Due to the aging population, the incidence and prevalence of degenerative conditions, such as PD, have increased in the last decades. Motor and autonomic symptoms and neuropsychiatric complications increase as the condition progresses [8]. Dementia and depression are associated with disease severity and mortality in PD [9].

The Spanish centralized hospital discharge database, which covers most of the Spanish population and includes more than 98% of hospital admissions in the national health care system, provides a complete record of all hospitalizations and is generally not subject to the limitations of outpatient surveillance systems. This database has been used for research purposes, including epidemiological studies on infectious diseases, anaphylaxis or cancer [10–12]. Similar databases have been used to measure Parkinson’s disease hospitalizations in other European countries [13].

The objective of this study was to estimate the incidence of hospitalizations associated with PD from 1997 to 2012 in Spain and to describe the epidemiology of these hospitalizations. Thus, this study compared hospitalizations associated with PD as a primary diagnosis versus hospitalizations associated with PD as an important comorbidity. It also investigated differences in terms of the temporal trend of hospitalization rates and disease severity, as estimated by the average length of stay and in-hospital case fatalities.

## Materials and Methods

A retrospective study using the national information system for hospital data [Conjunto Mínimo Básico de Datos (CMBD)] from the Ministry of Health was performed. This system contains information about the admission date, discharge date, age, sex, geographical region, diagnosis, clinical procedures, and discharge status (e.g., in-hospital death and recovery) for all hospitalizations in Spain. The CMBD uses clinical codes from the Spanish version of the 9th International Classification of Diseases (ICD-9-MC), and it includes an estimated 98% of public hospital admissions, covering 99.5% of the Spanish population [14]. We assumed that the remaining population and hospitalization information not included in the study followed the same epidemiological characteristics.

The unit of analysis were all hospital discharges related to Parkinson´s disease (ICD 9 CM: CIE-9: 332.0 paralysis agitans) that were listed in any diagnostic position reported for the general population over a 16-year period (January 1, 1997 through December 31, 2012). The first diagnostic position is the main cause of hospitalization.

For each case, specific data were gathered on age, sex and diagnostic position. The following patient age groups were established: younger than 30 years old, 30–40 years old, 40–50 years old, 50–60 years old, 60–70 years old, 70–80 years old, 80–85 years old and older than 85 years of age. The most frequent comorbidities and clinical procedures were studied using a subset of data that registered only those patients 50 years of age and older who were hospitalized with Parkinson´s disease in the first diagnostic position beginning in 2012. In the same subset, the main cause of hospitalization was examined when PD was coded in a secondary position.

### Statistical methods

The annual hospitalization rate (HR) for the entire population of Spain was calculated as the number of hospital discharges per 100,000 inhabitants. Data from the age- and sex-specific annual municipal population registries (corrected by the CMBD national coverage) were used as the denominator.

The average length of hospital stay (ALOS) was the number of hospitalization days averaged across all hospital discharges. The in-hospital case-fatality rate (CFR) was calculated as the number of deaths during the hospital stay divided by the total number of hospital discharges (%), and 95% confidence intervals were calculated. Binomial regression [generalized linear regression (GLM), with a log link and binomial distribution for the error], was used to assess differences in the hospitalization rates (per 100,000 inhabitants) by year, age group and sex. Differences in the proportions were assessed with the Chi-squared test. ANOVA and Kruskal-Wallis tests were used for multiple comparisons by year and age group.

The costs of these hospitalizations to the health care system are based on estimates from the Ministry of Health. The cost was calculated by considering the diagnostic cost group, total cost and number of discharges. The diagnostic cost group was based on the diagnosis related groups (DRG) for the hospitalized patients, which was based on the discharge ICD classification, age, sex, and resource consumption. DRG calculations were made using 3M Core Grouping System Software [15,16].

In all tests, the significance level was p < 0.05. Statistical analyses were performed using SAS University Edition and R Studio, version 3.0.3.

The present study received a waiver for informed consent from the local ethics committee (Comité de Ética de la Investigación de la Universidad Rey Juan Carlos). The patient information was anonymized and de-identified prior to the analysis.

## Results

### Hospitalizations in patients with PD in any diagnostic position

A total of 438,513 hospital discharges were reported for patients with PD during the 16-year study period. The mean patient age was 78.17 years (SD, 8.68), and 52% of the patients were male. The annual average cost of hospitalizations for patients with PD in the National Health Care System was € 120 M, with a mean hospitalization cost of €4,378 (SD, €3,643). Eighty-six percent of the patients were admitted from the emergency room.

**Fig 1:**
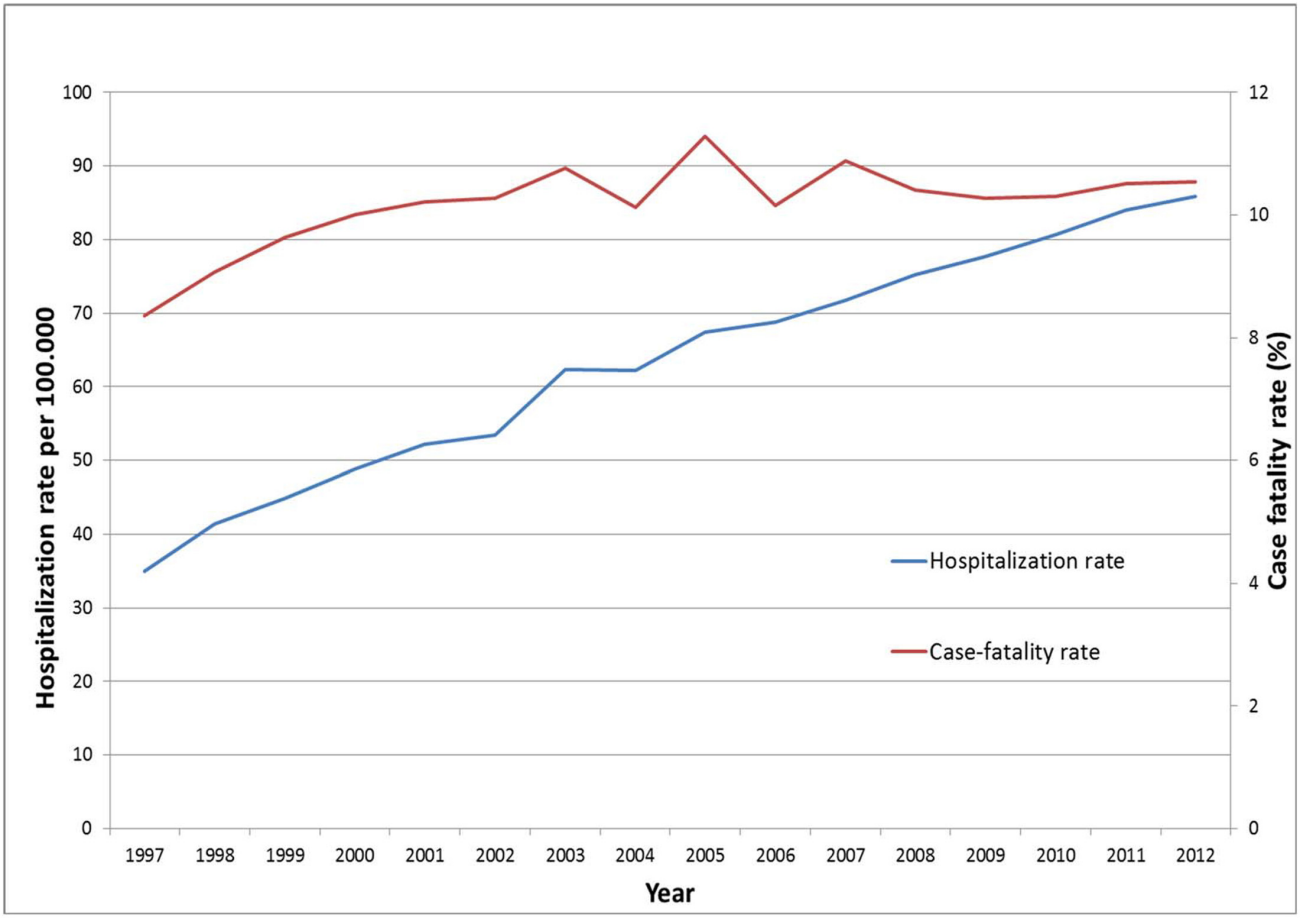
Hospitalization rate and case-fatality rate in patients with Parkinson´s disease in Spain (1997–2012).

The hospitalization rate (HR) was 64.19 (95% CI: 64.00–64.38) hospitalizations per 100,000 population. The average length of hospital stay significantly decreased (p<0.001) from 12.74 days (SD = 17.02 days) in 1997 to 8.97 days (SD, 9.99 days) in 2012. The average was 10.47 days (SD, 12.65 days). There were 45,216 in-hospital deaths during the study period, with a case fatality rate of 10.31% (95% CI: 10.22–10.40). Both HR and CFR significantly increased during the study period (HR_1997_ 34.91, 95% CI: 34.32–35.50) to HR_2012_ 85.85 (95% CI: 85.01–86.69), p_trend_ <0.001 and CFR_1997_ 8.36% (95% CI: 7.89–8.83) to CFR_2012_ 10.54 (95% CI: 10.24–10.85), p_trend_ <0.001, Fig 1)

The HR and CFR were higher in males, with a HR of 67.49 (95% CI: 67.21–67.77) vs. females 60.97 (95% CI: 60,71–61,23) hospitalizations per 100,000 inhabitants (p<0.001), and a CFR of 11.03% (95% CI: 10.90–11.16) in males vs. 9.54% (95% CI: 9.42–9.67) in females (p < 0.001). No differences in the length of hospital stay were observed between males and females.

In addition, a significant increase in HR (p < 0.001) and CFR (p <0.001) was observed by age group during the study (Table 1). The highest incidence of hospitalization was observed in patients older than 85 years of age, with a hospitalization rate of 749 (95% CI: 744–753) per 100,000 inhabitants.

**Table 1:** Hospitalization rate and case-fatality rate of patients with Parkinson´s disease by age group in Spain (1997–2012).

	Hospitalization Rate (per 100.000 inhabitants)		Case Fatality Rate (%)	
	Rate	95% CI	Rate	95% CI
< 30 years	0.12	0.10–0.13	1.76	0.23–3.29
30 years-40 years	0.60	0.56–0.65	0.15	0.00–0.43
40 years-50 years	2.71	2.61–2.81	1.45	1.00–1.90
50 years-60 years	12.52	12.27–12.77	2.69	2.38–3.01
60 years-70 years	69.61	68.97–70.25	4.85	4.66–5.05
70 years-80 years	317.49	315.98–319.00	8.34	8.21–8.47
80 years-85 years	672.89	668.93–676.84	11.84	11.65–12.03
>85 years	748.59	743.95–753.22	15.57	15.35–15.80
TOTAL	64.19	64.00–64.38	10.31	10.22–10.40

### Comparing hospitalizations in patients with PD as the main cause of hospitalization to patients with PD coded as a comorbidity

When restricting PD as the main cause of hospitalization (“primary PD”), a total of 23,086 hospital discharges were reported during the 16-year study period. The HR was 3.38 (95% CI: 3.34–3.42) hospitalizations per 100,000 population. The average length of hospital stay was 10.55 days (SD, 13.08 days). The mean patient age was 69.26 years (SD, 11.39 years), and 53% of the patients were male. The mean hospitalization cost per hospitalization was €4,839 (SD, €3,655). Eighty-six percent of the patients with primary PD were admitted from the emergency room.

During the study period, there were 571 deaths in patients with primary PD, with a case-fatality rate of 2.47% (95% CI: 2.27–2.67).

The HR and CFR were significantly higher in males. The HR was 3.64 (95% CI: 3.57–3.70) vs. 3.13 (95% CI: 3.3.07–3.19) hospitalizations per 100,000; p<0.001, and the CFR was 2.89% (95% CI: 2.60–3.19) vs. 2.00% (95% CI: 1.74–2.26); p<0.001 for males and females, respectively. No difference in the length of stay was observed between males and females.

The HR and CFR in patients with primary PD significantly increased with age (p<0.001 and p<0.001, respectively) but did not vary during the study period (p = 0.1632 and p = 0.3970, respectively). The average length of stay significantly decreased (p<0.0001) from 13.96 days (SD, 18.03 days) in 1997 to 7.76 days (SD, 10.41 days) in 2012.

For PD coded as a comorbidity (“comorbid PD”, a total of 415,427 discharges, the average HR was 64.19 hospitalizations (95% CI: 64.00–64.38) per 100,000 inhabitants. The average annual increase in hospitalizations associated with primary PD and comorbid PD differed (p = 0.001). The average annual increase in primary PD hospitalization was non-significant (1.14%, p = 0.397), while the average annual increase among comorbid PD hospitalizations was significant (15.37%, p = 0.001; Table 2).

**Table 2:** Hospitalization rate of patients with Parkinson´s disease in Spain (1997–2012).

Year	First Diagnostic Position (Main cause of hospitalization)		Secondary Diagnostic Position	
	Hospitalization Rate (per 100,000 inhabitants)	95% CI	Hospitalization Rate (per 100,000 inhabitants)	95% CI
1997	3.06	2.88–3.23	34.91	34.32–35.50
1998	3.39	3.21–3.58	41.46	40.82–42.10
1999	3.17	2.99–3.35	44.87	44.21–45.53
2000	3.14	2.96–3.31	48.89	48.20–49.58
2001	3.46	3.28–3.64	52.12	51.41–52.82
2002	3.27	3.09–3.44	53.46	52.75–54.17
2003	3.55	3.37–3.73	62.35	61.59–63.10
2004	3.54	3.36–3.72	62.25	61.50–63.00
2005	3.32	3.15–3.49	67.41	66.63–68.18
2006	3.35	3.18–3.52	68.76	67.98–69.54
2007	3.33	3.16–3.50	71.70	70.91–72.49
2008	3.65	3.48–3.83	75.28	74.48–76.08
2009	3.25	3.09–3.42	77.64	76.83–78.45
2010	3.43	3.26–3.60	80.62	79.80–81.44
2011	3.45	3.28–3.61	84.04	83.20–84.87
2012	3.62	3.45–3.80	85.85	85.01–86.69
TOTAL	3.38	3.34–3.42	64.19	64.00–64.38

**Fig 2:**
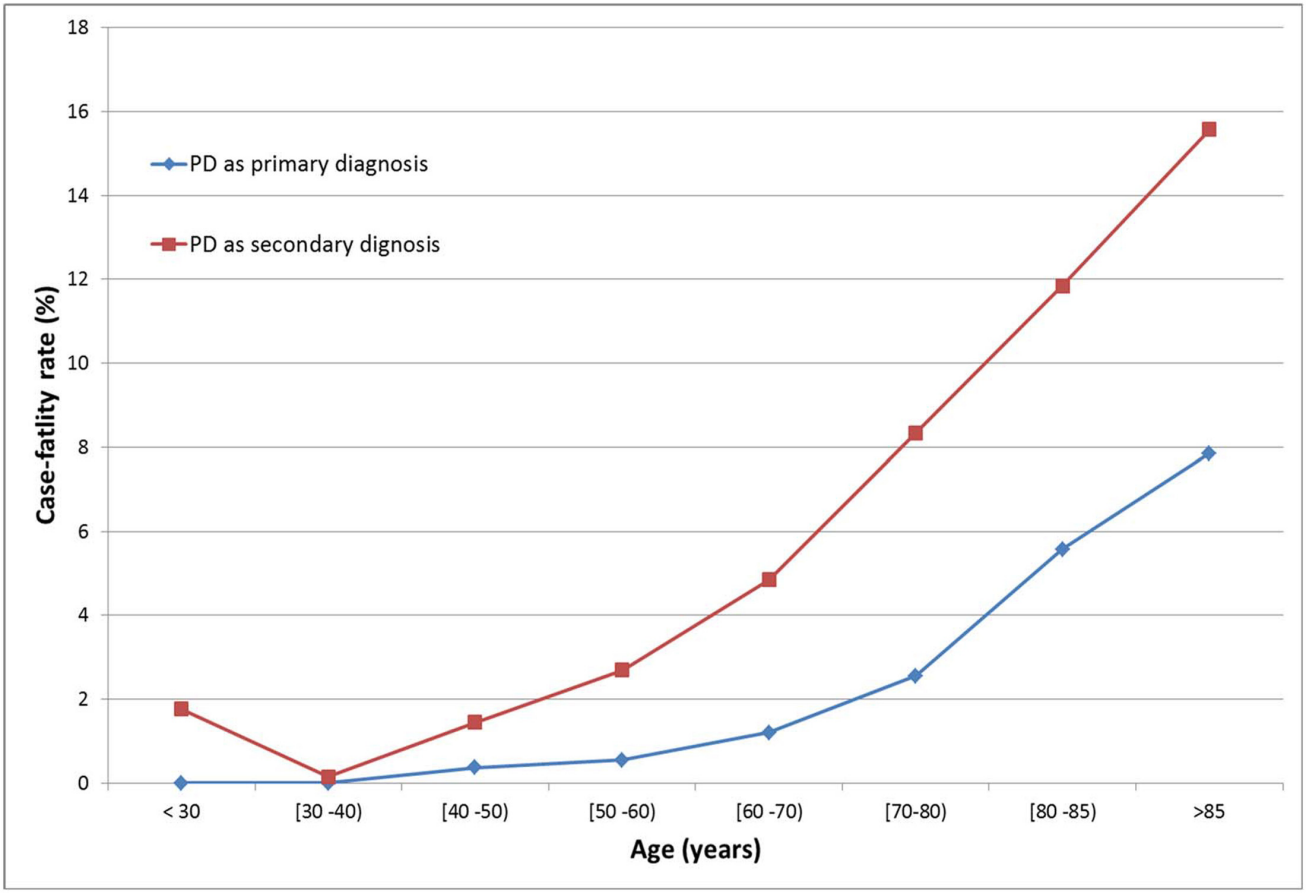
Case fatality rate of patients with PD as the primary diagnosis (cause of hospitalization) and as the secondary diagnosis in Spain (1997–2012).

Fig 2 shows the differences in in-hospital CFR between the primary PD hospitalizations and comorbid PD hospitalizations. The average in-hospital case-fatality rate among patients with comorbid PD was 10.31 (95% CI: 10.22–10.40).

On average, the hospitalized patients with comorbid PD were older (78.17 years of age; SD, 8.68 years), while the proportion of males was similar to that of the patients with primary PD (52%). Likewise, no difference in the length of stay was observed between the primary PD and comorbid PD hospitalizations.

### Most frequent comorbidities and procedures and causes of hospitalizations

In 2012, a total of 41,726 discharges occurred among patients with PD in any diagnostic position who were 50 years of age and older.

Of the 1,765 patients hospitalized due to primary PD, 87% had at least one co-morbidity recorded. The most frequent comorbidities were unspecified essential hypertension, n = 593 (34%); diabetes mellitus type II, n = 266 (15%); unspecified hyperlipidemia, n = 239 (14%); depressive disorder not classified elsewhere, n = 143 (8%); atrial fibrillation, n = 130 (7%); and urinary tract infection site not specified, n = 128 (7%).

In patients with PD, the most frequently occurring advanced therapies and procedures were a) implantation or replacement of intracranial neurostimulator lead(s) n = 209 (12%) and b) percutaneous (endoscopic] gastrostomy (PEG) n = 121 (7%).

Among the 39,961 hospital discharges with comorbid PD, the most frequent causes of hospitalization were (Table 3) respiratory system diseases, n = 9649 (24%); circulatory system diseases, n = 7471 (19%); injuries and poisoning, including fractures, n = 4721 (12%); digestive system diseases, n = 4077 (10%) and neoplasms n = 2048 (5%).

**Table 3:** Causes of hospitalization when PD was not coded as the primary diagnosis*.

ICD9 CODE	n	%
1. Infectious and parasitic diseases (001–139)	1375	3.44
2. Neoplasms (140–239)	2048	5.12
3. Endocrine, nutritional and metabolic diseases, and immunity disorders (240–279)	860	2.15
4. Diseases of the blood and blood-forming organs (280–289)	375	0.94
5. Mental disorders (290–319)	997	2.49
6. Diseases of the nervous system and of the sense organs (320–389)	981	2.45
7. Diseases of the circulatory system (390–459)	7471	18.70
8. Diseases of the respiratory system (460–519)	9649	24.15
9. Diseases of the digestive system (520–579)	4077	10.20
10. Diseases of the genitourinary system (580–629)	3214	8.04
12. Diseases of the skin and subcutaneous tissue (680–709)	455	1.14
13. Diseases of the musculoskeletal system and connective tissue (710–739)	1170	2.93
14. Congenital anomalies (740–759)	24	0.06
16. Symptoms, signs, and ill-defined conditions (780–799)	1891	4.73
17. Injuries and poisoning (800–999)	4721	11.81
Supplementary classification of factors influencing health status and contact with health services (V01-V89)	618	0.316
Missing (ZZZ.ZP)	35	0.088
TOTAL	39961	100

* These hospitalization causes were obtained in 2012 in Spain from all hospitalizations without PD in the first diagnostic position among patients 50 years of age and older.

## Discussion

In this retrospective study of all PD-associated hospital discharges in Spain, we identified an average annual increase of 15% in the number of hospitalizations listing PD as a comorbid condition, while the number of hospitalizations listing PD as the main cause remained constant.

As expected, we found that the hospitalization rates in patients with PD increased with age [17]. Likewise, the differences between males and females were consistent with recent studies reporting a higher incidence of PD among men in the general population [18], although some older studies [19, 20] have not observed significant differences by sex. Similar to other studies, the most frequent reasons for hospitalization were respiratory infections, urinary tract infections, cardiovascular diseases, falls and fractures [21,13,22,23].

Hospitalizations associated with PD in any diagnostic position increased by 2.5-fold during the 16-year observational period in Spain. Our results show that 95% of these hospitalizations were due to a comorbid disorder. This percentage exceeds that described by Braga et al., who found that the presence of a comorbid disorder caused 80% of all hospitalizations in PD patients [21].

Moreover, our study observed an average annual increase of 15% in the rate of PD patients hospitalized because of important comorbidities. This rate compares to a non-significant 1.14% annual increase in the rate of primary PD hospitalizations and to an annual increase of 1.23% in the rate of hospitalizations due to any cause (http://www.msssi.gob.es/estadEstudios/estadisticas/cmbd.htm), most likely due to population aging. These results indicate that although deterioration of PD during hospitalization has been widely acknowledged [24], this disproportional increase in comorbid PD hospitalizations poses a challenge to clinicians who lack PD specific knowledge.

Increased awareness of the added risk PD poses in acute hospitalized patients can be used to develop strategies to improve patient outcomes [23]. Regular neurologist care in PD is specifically associated with a lower risk of hospitalization and re-hospitalization, and deficiencies in the in-hospital prescribing of medications for PD patients have been reported [25]. It is, therefore, important to inform the hospital staff outside of the neurology units about the importance of adhering to outpatient medication schedules and to provide specific training to identify medications that worsen PD, with the goal of reducing important complications, such as those related to reduced mobility and aspiration pneumonia. [26,27]

It is difficult to interpret differences in the case fatality rates between hospitalizations with PD as the main cause and hospitalizations with PD as a comorbid condition because the main cause of hospitalization and the associated severity of the latter patient group are heterogeneous. One explanation might be the difference in age. On average, patients hospitalized for other causes but with PD as a comorbidity have a higher intrinsic mortality risk than those hospitalized with primary PD because they are, on average, ~9 years older.

The average length of stay of 10.5 days in our study is similar to that of other published studies in Europe [21], but it is higher than the 7 days reported in Australia [22]. While the average length of stay, a common measure of severity, did not differ between primary and comorbid PD hospitalizations, the overall length of stay decreased by 2.8 months per year during the observational window. It is difficult to attribute this reduction to changes in care specific to PD because the reduction was similar to the decrease of 2.5 months per year reported for all-cause hospitalizations by the Spanish Ministry of Health (http://www.ine.es/jaxi/menu.do?type=pcaxis&path=/t15/p414&file=inebase&L=0).

Hospitalization of patients with PD costs the National Health Care System 120 million € each year. The financial impact of PD on society has been discussed broadly in the last decades, and it is expected to increase in the future, along with the aging population [5]. Although these costs cannot be attributed solely to PD, it is more complicated to manage these patients, and the average length of hospitalization is increased [13]. A prospective study in the US reported that one-third of people with PD had a hospital encounter each year [28]. In addition, the cost of PD care increases with the disease in progression [5, 29].

This study has some limitations derived from the use of the CMBD. The CMBD only records hospitalized cases; therefore, our results cannot be taken as incidence or prevalence rates. Ninety-five percent of the reported hospitalizations were in patients with PD in a secondary diagnostic position; PD was the main cause of hospitalization in only 5% of the patients. Our results cannot be interpreted as a state of the epidemiology of PD but as a measure of the hospital burden in patients with PD. The reliability of the CMBD depends on the quality of the discharge report and the clinical histories, as well as the codification process variables. Quality controls have been performed to assess the validity of the CMBD, and the codification process has improved since 2001 [16], making CMBD a useful tool for this approach.

In conclusion, this study revealed an important increase in hospitalizations with Parkinson´s disease as a comorbid condition but not as the primary cause of hospitalization. In an aging population with an increasing prevalence of neurodegenerative disorders, an important effort is needed to improve the management of these multimorbid patients, particularly in the hospital setting.
